# Successful Treatment of Myasthenia Gravis Following PD-1/CTLA-4 Combination Checkpoint Blockade in a Patient With Metastatic Melanoma

**DOI:** 10.3389/fonc.2019.00084

**Published:** 2019-02-14

**Authors:** Jan-Michael Werner, Viola Schweinsberg, Michael Schroeter, Boris von Reutern, Michael P. Malter, Max Schlaak, Gereon R. Fink, Cornelia Mauch, Norbert Galldiks

**Affiliations:** ^1^Department of Neurology, University of Cologne, Cologne, Germany; ^2^Department of Dermatology, University of Cologne, Cologne, Germany; ^3^Center of Integrated Oncology, University of Cologne, Cologne, Germany; ^4^Institute of Neuroscience and Medicine (INM-3), Forschungszentrum Juelich, Juelich, Germany

**Keywords:** nivolumab, ipilimumab, myasthenic crisis, immunotherapy, checkpoint inhibitor, neuro-oncology

## Abstract

Currently, the blockade of certain immune checkpoints such as the cytotoxic T-lymphocyte-associated protein 4 (CTLA-4) and programmed cell death-1 (PD-1) using checkpoint inhibitors is standard of care in patients with metastatic melanoma, especially with BRAF wild-type. However, several checkpoint inhibitor-related complications have been reported, including severe adverse events in the central and peripheral nervous system. In particular, in the recent past, the occurrence of myasthenia gravis following checkpoint inhibitor monotherapy, particularly nivolumab or ipilimumab, has been reported. In contrast, reports on PD-1/CTLA-4 combination blockade—usually with fatal clinical outcome—are scarce. We here report a case with combination immune checkpoint blockade-related myasthenia gravis with favorable clinical outcome.

## Background

The introduction of immunotherapy, particularly the blockade of immune checkpoints such as the cytotoxic T-lymphocyte-associated protein 4 (CTLA-4) and programmed cell death receptor-1 (PD-1) using checkpoint inhibitors, has resulted in a significant improvement of prognosis and overall survival of melanoma patients. The blockade of CTLA-4 using the checkpoint inhibitor ipilimumab was the first breakthrough in this field leading to an improved overall survival in this group of patients (1). Furthermore, in patients with metastatic melanoma without BRAF mutation, treatment with the checkpoint inhibitor nivolumab targeting PD-1 improved the overall and progression-free survival compared to conventional chemotherapy (2). More recently, it has been reported that the combination of nivolumab with ipilimumab seems to be more effective than checkpoint inhibitor monotherapy in patients with untreated melanoma (3) suggesting that the combination of checkpoint inhibitors may play a more significant role in melanoma therapy in the future.

However, the increased use of checkpoint inhibitors for anticancer treatment has also led to an increased occurrence of different immune-related adverse events (irAE) (4). In particular, irAEs may affect the central and peripheral nervous system resulting in severe neurological complications including encephalitis, aseptic meningitis, transverse myelitis, meningoradiculitis, hypophysitis, Guillain-Barré syndrome and its variants, peripheral neuropathy, and myasthenia gravis (MG) (5).

MG is a disorder that affects neuromuscular transmission. It is currently one of the best characterized autoimmune disorders. The cardinal feature of MG is an exercise-induced weakness of ocular muscles, bulbar functions, as well as limb and respiratory muscles, which is due to an immune attack against postsynaptic structures of the neuromuscular junction.

Regarding immunotherapy-related MG (irMG), a retrospective study analyzed data from 2014 to 2016 of more than 9,800 patients treated with nivolumab, and ~400 patients with ipilimumab (6). Overall, neurological irAE occurred in 6% of the patients treated with nivolumab, and 9% undergoing ipilimumab therapy. Interestingly, no patient treated with ipilimumab developed an irMG, whereas 8% of the patients treated with nivolumab that experienced neurological irAE developed an irMG (6). Thus, in that study, the overall prevalence of irMG following nivolumab was 0.1% (12 of 9,869 patients) (6). Importantly, compared to patients developing MG independent from immunotherapy, patients with irMG seem to have a higher rate of potentially life-threatening myasthenic crises necessitating respiratory support (6–8).

## Case Presentation

We here report on the development of an irMG following PD-1/CTLA-4 combination immune checkpoint blockade for the treatment of a metastatic melanoma, which has hitherto not been described. Twelve months after the initial diagnosis of a melanoma of the right lower leg (Clark-Level III, tumor thickness of 2.4 mm), a 62-year-old male patient without BRAF mutation developed lymphogenic metastases and was consecutively treated systemically using the combination of nivolumab (1 mg/kg body weight every 3 weeks) plus ipilimumab (3 mg/kg body weight every 3 weeks).

**Figure 1:**
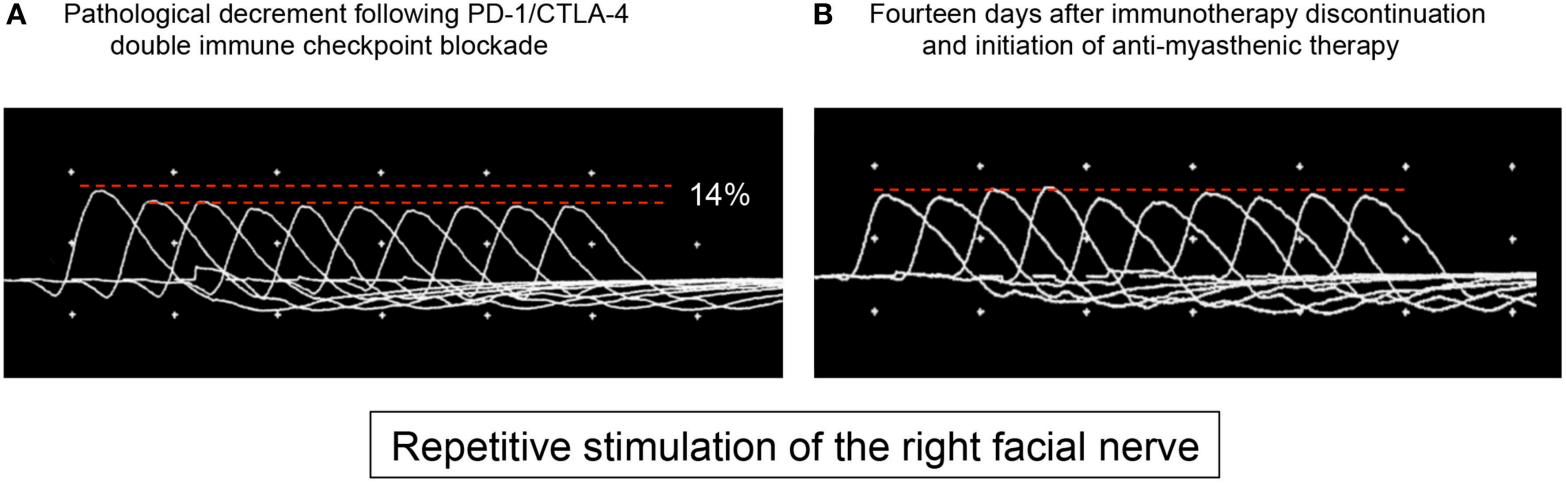
At admission **(A)**, the patient presented with ptosis of the right eye, and repetitive stimulation of the right facial nerve revealed neuromuscular dysfunction with a decrement of 14%. Two weeks after discontinuation of immunotherapy and treatment with pyridostigmine and prednisone, the patient showed no clinical signs of myasthenia and no pathological decrement could be detected **(B)**.

Four weeks after treatment initiation, the patient suffered from fatigue and developed ptosis of the right eye. On clinical examination, the Simpson test of the right eye was positive after 30 s. No other MG-related symptoms such as general muscle weakness or dysarthria could be objectified (MGFA class I). The further neurological examination was also regular. Contrast-enhanced MRI of the brain showed no disruption of the blood-brain barrier, especially no leptomeningeal enhancement. Analysis of the cerebrospinal fluid revealed a slightly elevated leukocyte cell count (13 cells/μl) with the presence of erythrocytes, most probably due to a traumatic lumbar puncture. Cytopathology of the cerebrospinal fluid showed no tumor cells. Repetitive stimulation of the right facial nerve revealed an action potential decrement of 14% (Figure 1A). Autoantibodies against muscle-specific tyrosine kinase, acetylcholine receptors, and titin could not be detected serologically. Consequently, a grade 1 irMG was considered (4) and the checkpoint inhibitor combination was discontinued. Regarding the irMG, the patient was treated with pyridostigmine (300 mg/d) and prednisone (20 mg/d). Hereby, we differed from the frequently suggested dosage of 1 mg/kg prednisone daily as this dosage may lead to transient clinical deterioration (and potential respiratory failure) in up to 50% of cases and our patient presented only mild clinical symptoms (5). Already after 2 days, ptosis and fatigue started to improve. After 14 days, the Simpson test was negative. Repetitive nerve stimulation was repeated, and pathological decrement could not be observed any longer (Figure 1B). Within 6 weeks, all neurological symptoms recovered completely and nivolumab monotherapy was restarted. The patient received three cycles (3 mg/kg body weight every 3 weeks), followed by a fixed dose (480 mg) every 4 weeks, and pyridostigmine was tapered and subsequently discontinued. During a follow-up of 2 months, no symptoms of MG occurred. A written informed consent was obtained from the patient for publication of this case report.

## Conclusion

Predominantly from 2016 to 2017, reports on irMG have been added to the literature. Following treatment with anti-PD-1 inhibitor monotherapy (i.e., nivolumab or pembrolizumab), Makarious and colleagues summarized 12 patients with metastatic melanoma or other extracranial primary tumors (e.g., non-small cell lung cancer, squamous cell carcinoma of the bladder, renal cell carcinoma) who developed an irMG (9). Regarding anti-CTLA-4 inhibitor-induced MG, four case reports of metastatic melanoma patients described the occurrence of irMG following ipilimumab monotherapy (9). Furthermore, checkpoint inhibitor monotherapy may also exacerbate preexisting MG (9).

To the best of our knowledge, data on neurological irAE, in particular, irMG, following combination immune checkpoint blockade are extremely rare. Loochtan and colleagues reported in 2015 a patient with an extensive stage small cell lung cancer, who developed an irMG during combined PD-1 and CTLA-4 checkpoint inhibitor blockade (nivolumab and ipilimumab) and subsequently died due to myasthenia-associated complications (7). One year later, Antonia et al. described a patient with advanced non-small cell lung cancer in whom the combination of durvalumab, an antibody directed against the immune checkpoint programmed cell death-ligand 1 (PD-L1), and tremelimumab (newer generation monoclonal antibody against CTLA-4) caused a severe irMG with myasthenia-associated mortality (8). Last year, Chen and coworkers reported a patient with a squamous cell carcinoma of the lung who developed an irMG, myositis and polyneuropathy following combination checkpoint inhibitor blockade (nivolumab and ipilimumab) (10). Subsequently, that patient died due to complications related to a severe weakness of respiratory muscles.

In contrast to these three reported cases in lung cancer patients with fatal clinical course following combination immune checkpoint blockade despite extensive anti-myasthenic therapy, we report for the first time a relatively mild form of irMG without fatal outcome in a patient with metastatic melanoma treated with nivolumab plus ipilimumab. Importantly, our patient improved clinically after discontinuation of immunotherapy and the initiation of anti-myasthenic therapy, which is in contrast to the reported case by Loochtan et al. (7). Moreover, our patient tolerated the restarted nivolumab monotherapy, which is in contrast to the other cases reported so far. However, the reasons for the mild clinical course and response to irMG treatment in our case are unclear. It is therefore tempting to speculate whether the early discontinuation of immunotherapy and subsequent anti-myasthenic therapy may have prevented a possible severe progression of irMG, i.e., myasthenic crisis.

## Concluding Remarks

The increasing use of checkpoint inhibitors and combinations thereof requires the interaction of neurologists, neurooncologists as well as medical oncologists to recognize the full spectrum of irAE including irMG at an early stage, particularly regarding their potential severity and the necessity to react promptly.

## Ethics Statement

The patient gave written informed consent in accordance with the Declaration of Helsinki. The patient's personal identifiers were not included in this manuscript.

## Author Contributions

J-MW, MSchr, and NG: Study concept and design. VS and BvR: Acquisition of data. J-MW and NG: Analysis and interpretation. J-MW and NG: Manuscript drafting. MSchr, MPM, MSchl, GRF, and CM: Critical revision of the manuscript for important intellectual content. NG: Study supervision.

### Conflict of Interest Statement

MSchr received speaker's honoraria from Alexion, Biogen, Genzyme/Sanofi, Grifols, Miltenyi, and Roche. The remaining authors declare that the research was conducted in the absence of any commercial or financial relationships that could be construed as a potential conflict of interest.
